# Characterization of Cu(II)-ACC Complexes and Conversion of the Bound ACC into Ethylene in the Presence of Hydrogen Peroxide. Detection of a Brown Intermediate at Low Temperature

**DOI:** 10.1155/2007/43424

**Published:** 2007-09-10

**Authors:** Wadih Ghattas, Michel Giorgi, Christian Gaudin, Antal Rockenbauer, Marius Réglier, A. Jalila Simaan

**Affiliations:** ^1^BiosCiences FRE CNRS 3005, Faculté des Sciences et Techniques, Université Paul Cézanne Aix-Marseille III, avenue Escadrille Normandie-Niémen, 13397 Marseille Cedex 20, France; ^2^Spectropôle, Faculté des Sciences et Techniques, Université Paul Cézanne Aix-Marseille III, avenue Escadrille Normandie-Niémen, 13397 Marseille Cedex 20, France; ^3^Central Research Institute for Chemistry, Hungarian Academy of Sciences, P.O. Box 17, 1525 Budapest, Hungary

## Abstract

Two copper(II)-ACC complexes were prepared and characterized: [Cu(bpy)(ACC)(${\text{H}}_{2}\text{O}$)]⋅ ${\text{CO}}_{4}$ (1) and ${\left[\text{Cu}{\left(\text{ACC}\right)}_{2}\right]}_{3}$ ⋅ $4{\text{H}}_{2}\text{O}$ (2). Their crystallographic structures are described and analyzed. Spectroscopic characterizations (UV-visible and EPR) confirm that the structure is maintained in solution. These complexes are able to produce ethylene in the presence of hydrogen peroxide in an “ACC Oxidase-like” reaction in water and in methanol. The conversion of ACC into ethylene depends on the amount of base, and, in methanol, 3 equivalents of NaOH are needed for optimum activity. The base is proposed to play a role in ${\text{H}}_{2}{\text{O}}_{2}$ deprotonation. The presence of an exogenic ligand (bpy) is important for the reactivity and may stabilize a reaction intermediate. Indeed, a brown intermediate with an absorption band centered at 433 nm can be detected at low temperature when 1 is treated with 10 equivalents of ${\text{H}}_{2}{\text{O}}_{2}$.

## 1. INTRODUCTION

 Ethylene is a hormone that regulates many aspects of plant growth and development including senescence, germination as well as fruit ripening [1, 2]. The final step of ethylene biosynthesis is catalyzed by ACC Oxidase (ACCO), a mononuclear nonheme ferrous enzyme. ACCO catalyzes the two-electrons oxidation of 1-aminocyclopropane-1-carboxylic acid (ACC) in the presence (in vitro) of dioxygen and ascorbate to give ethylene, cyanide, and carbone dioxide (Sheme 1).

The first crystallographic structure of ACCO from *Petunia hybrida* has recently been obtained by the group of C; see Schofield [3]. This structure reveals that the active site contains a single iron(II) ion bound to the side chain residues of two histidines and an aspartate. The role of the metal ion in the reaction remains unclear. It is proposed that the first step consists in the fixation of O_2_ and the substrate on the Fe(II) ion [4–6]. The substrate is proposed to be coordinated to the iron in a bidentate mode *via* the nitrogen and one oxygen of the amine and the carboxylate groups, respectively. Few spectroscopic data on ACCO are available, however the following steps of the reaction could involve intermediates such as iron-peroxo or iron-oxo species in activation of the substrate [7, 8]. Thanks to stereochemical studies carried out with various substrates [9, 10], it was proposed that conversion of ACC into ethylene proceeds *via* a radical mechanism with the formation of an aminyl radical. A possible catalytic mechanism is presented on Sheme 2.

As we previously discussed, it is proposed that the substrate coordinates the metal ion in a bidentate mode. There are few data available in the literature concerning ACC coordination to metal ions. Moreover, there are only few reported metal complexes that are able to convert ACC into ethylene in an ACCO-like activity. In 1985, the group of J. Baldwin reported the oxidation of ACC into ethylene by several transition metal oxidants such as copper(II), permanganate, and ferrate ions in aqueous solution [10]. The group of Y. Nishida then studied the reactivity of several binuclear metal complexes (Mn(II), Fe(III), Co(II)) based on dinucleating ligands [11, 12]. In the presence of a large excess of hydrogen peroxide, these complexes are able to convert ACC into ethylene. The conversion yields remained however extremely low (especially in the case of the iron complexes) and no metal-ACC interaction was identified.

Spectroscopic and structural data on metal-ACC complexes are thus of great interest as well as reactivity studies. Many copper-aminoacid complexes were reported and crystallized [13]. Thereby, as a first approach, we focused on the interaction between a copper(II) ion and ACC. We recently reported the synthesis, the X-Ray structure and the reactivity of [(bpy)${\text{Cu}}^{\text{II}}$(ACC)(H_2_O)] · ClO_4_ complex (**1**) where bpy stands for bipyridine [14]. This complex was the first example of well-characterized metal-ACC adduct and was able to convert the bound ACC into ethylene in the presence of hydrogen peroxide. Here, we describe a more detailed study of this complex as well as the comparison with another copper(II)-ACC complex: [Cu(ACC)_2_ ]_3_ · 4H_2_O (**2**). Soon after its characterization in our laboratory, Judas and Raos [15] published a complete structural analysis of the latter complex (complex **2**). We will present here the reactivity studies on **2** and compare the structural and spectroscopic data for the two complexes.

## 2. EXPERIMENTAL SECTION

Commercially available chemicals were purchased and used without further purification. *Caution: perchlorate salts are potentially explosive and should be handled with care*.

**Synthesis of [(bpy)Cu(ACC)(H_2_O)] · ClO_4_ (1)**

The complex was prepared following a previously described procedure [14]. 10.1 mg of ACC (0.1 mmoles) in water was deprotonated by one equivalent of NaOH then added on a methanolic solution of Cu(ClO_4_)_2_ · 6H_2_O (37 mg, 0.1 mmoles). 15.6 mg of 2.2′-bipyridine (0.1 mmoles) in methanol was then added on the resulting solution. After 15 minutes stirring, a blue complex was precipitated upon addition of diethylether and collected by filtration (yield = 80%). Crystals suitable for X-Ray diffraction measurements were obtained by slow diffusion of diethylether into a methanolic solution of **1**. ${\text{C}}_{14}{\text{H}}_{16}{\text{N}}_{3}{\text{CuClO}}_{7}\;\text{M}=437.29\;\text{g}\cdot {\text{mol}}^{-1}$. Elementary analysis: C 38.33, H 3.93, N 9.60 (calculated: C 38.45, H 3.69, N 9.61). UV-visible (MeOH): λmax=600 nm, $\varepsilon =98\;{\text{mol}}^{-1}\cdot \text{L}\cdot {\text{cm}}^{-1}$.

**Synthesis of [Cu(ACC)_2_]_3_ · 4H_2_O (2)**

20.2 mg of ACC (0.2 mmoles) in water was deprotonated by one equivalent of NaOH then added on a methanolic solution of Cu(ClO_4_)_2_ · 6H_2_O (37 mg, 0.1 mmoles). A blue powder precipitates (yield = 60%). Crystals were obtained by evaporation of a methanolic solution of **1**. λmax=606 nm, $\varepsilon =117\;{\text{mol}}^{-1}\cdot \text{L}\cdot {\text{cm}}^{-1}$.

### 2.1. X-ray diffraction measurements

All crystals were mounted on glass fibers. Data were collected on a Bruker-Nonius KappaCCD diffractometer at 293 K. Structures were solved using SIR92 and refinement calculations were performed using SHELX-97. Crystal structure for complex **1** has been described previously [14].

*Crystal data for* **1**: C_14_H_16_N_3_O_7_ClCu, Mw = 437.29, monoclinic, blue crystal (0.4 × 0.2 × 0.2 mm^3^), a = 12.0940(1) Å, b = 19.5096(3) Å, c = 7.5502(2) Å, *β* = 102.8066(7)^°^, V = 1737.14(6) Å^3^, space group P2_1_/c, Z =4, *ρ* = 1.672 g·cm^−3^, *μ*(Mok*α*) = 14.54 cm^−1^, 15442 reflections measured in the 2.71–27.48^°^ *θ* range, 3586 unique (Rint = 0.034), 251 parameters refined on F^2^ to final indices R[${\text{F}}^{\text{2}}>4\sigma {\text{F}}^{\text{2}}$: 3265 reflections] = 0.0441, wR[3586 reflections] = 0.1022 $\left[w=1/[\sigma^{2}\left({\text{Fo}}^{2}\right)+{\left(0.0394\text{P}\right)}^{2}+2.0199\text{P}\right]$ where $\text{P}=\left({\text{Fo}}^{2}+2{\text{Fc}}^{\text{2}}\right)/3]$. All hydrogen atoms were found experimentally, included into the calculations but not refined. The perchlorate anion was found to be disordered and the oxygen atoms were split and refined on several sites. The final residual Fourier positive and negative peaks were equal to 0.461 and −0.523, respectively. CCDC 288376.

*Crystal data for* **2**: see [15].

### 2.2. UV-visible spectroscopy

UV-visible spectra were recorded on a VARIAN Cary 50 probe spectrometer equipped with an HELLMA low temperature probe (TO 5 mm, ref 661202UV-5).

Low temperature measurements were performed using an ethanol/liquid N_2_ bath at the desired temperature.

### 2.3. EPR spectroscopy

EPR spectra were obtained using a BRUKER EMX9/2.7 spectrometer equipped with a digital temperature controller B-VT2000 (100–400 K).

The simulations with automatic parameter fitting were performed for rhombic symmetry [16]. The contribution of naturally abundant ^63^Cu and ^65^Cu was considered, but the values given in the text refer to ^63^Cu.

### 2.4. Ethylene production

The general procedure for activity assays was the following: 1 mL of a 1 mM solution of complex **1** or **2** or of a standard solution composed of an equimolar quantity of Cu(ClO_4_)_2_ · 6H_2_O and ACC was placed into a 16 mL hermetically sealed tube with or without NaOH. ten equivalents. of H_2_O_2_ was added through the septum. After one hour, 0.5 mL of gas from the head space was removed using a gas-tight syringe and analyzed by Gas Chromatography using a CHROMPACK CP 9002 gas chromatograph equipped with a POROPAK Q 80/100 column (1/8″). The following conditions were used: vector = N_2_, ${\text{T}}_{\text{injector}}\;=\;150^{{}^{\circ}}$C, ${\text{T}}_{\text{oven}}\;=\;80^{{}^{\circ}}$C, ${\text{T}}_{\text{detector}}\;=\;250^{{}^{\circ}}$C. The quantity of ethylene was quantified *versus* an external standard (Alltech 1% Ethylene in Nitrogen).

## 3. RESULTS

### 3.1. X-ray structures

Complexes **1** and **2** have been prepared as blue crystals from slow evaporation of methanol solutions. Complex **1** has been characterized by single crystal X-Ray diffraction technique [14]. It crystallizes in a monoclinic system and it was solved in the space group P2_1_/c. We also obtained crystals of complex **2** that have been analyzed by X-Ray diffraction technique. However, the same structure was, shortly after, described by Judas and Raos [15]. In Table 1, selected crystallographic data are reported for the two structures as obtained in our group.

**Figure 1:**
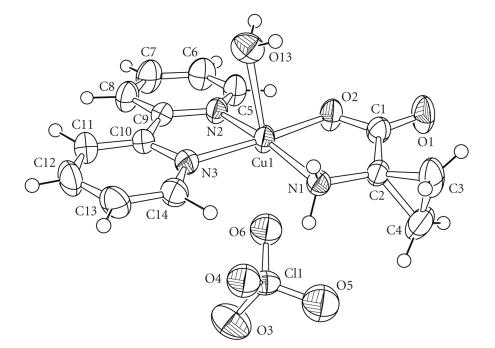
ORTEP drawing of **1**.

Figure 1 shows the ORTEP drawing of **1**. It reveals that the copper(II) ion is in pseudo-octahedral geometry. The ACC and the bpy ligands form the basal plane and a water molecule is coordinated to the Cu(II) on elongated axial position. Moreover, a disordered perchlorate anion was found close to the copper and refined on three different sites (only one position is shown). One oxygen atom of each of the three species is standing at a distance ranging from 2.85 to 2.99 Å from the copper (especially in the case of a minor site: a perchlorate oxygen is located at ca. 2.85 Å from Cu). Table 2 presents selected bond lengths and angles for **1**. The ACC ligand is coordinated *via* the nitrogen atom (d = 2.005 Å) and *via* one oxygen atom from the carboxylate function (d = 1.916 Å) as proposed in the ACC Oxidase's catalytic cycle. These bond lengths are in agreement with the average Cu(II)-aminoacid distances for complexes of similar geometry [13]. Indeed, analysis of the Cambridge data base reveals average distances ranging from 1.96–2.03 Å for Cu−N and from 1.91–1.97 Å for Cu−O bonds. The bpy ligand is coordinated to the copper ion at distances of Cu−N = 1.98 and 2.01 Å. The water molecule stands on the elongated axial position at 2.42 Å. Within the ACC moiety, the C−C distance C3−C4 is found at ca. 1.48 Å. This value is shorter than the similar value found in free ACC molecule for which several X-ray structures have been obtained [17, 18]. Indeed, the similar C−C distance is found from 1.490 to 1.497 Å in unbound ACC. A similar observation is made with M(II)-ACC pyridoxal Schiff base complexes (M = ${\text{Cu}}^{\text{II}}$ and ${\text{Ni}}^{\text{II}}$) [19, 20]. This is accompanied by a slight closure of the C3−C2−C4 angle (found at 59.2 Å *versus* values ranging from 59.6 to 59.8 in unbound ACC).

**Figure 2:**
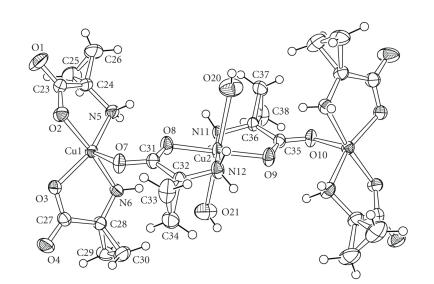
ORTEP drawing of **2**.

The structure of **2** is presented on Figure 2. It has been extensively described by Judas et al. [15]. It consists in an original arrangement where the asymmetric unit is composed of a trimeric assembly of Cu(ACC)_2_ complexes. In the central complex, the two ACC are coordinated in *trans* configuration. The metal is weakly bound to two water molecules on the axial positions (at distances of 2.48 and 2.57 Å) providing a pseudo-octahedral geometry at the copper ion. In the external complexes, the two ACC ligands adopt a *cis* geometry and the axial position is occupied by the second oxygen atom from the carboxylate function of an ACC belonging to the central unit. The external coppers are thus in a distorted square pyramidal geometry. Selected bond lengths and angles are displayed in Table 3. The Cu−N and Cu−O distances are within the range of expected distances as well for the *trans* and for the *cis* complexes. A shortening of the C−C distance within the ACC moieties can also be observed and can be related to the closure of the angle around the *α* atom.

### 3.2. Spectroscopic characterization in solution

The UV-visible spectra of **1** or **2** in methanol present a d-d transition centered at 600 nm and 606 nm, respectively (with *ε* = 98 and 117 M^−1^ · cm^−1^).

**Figure 3:**
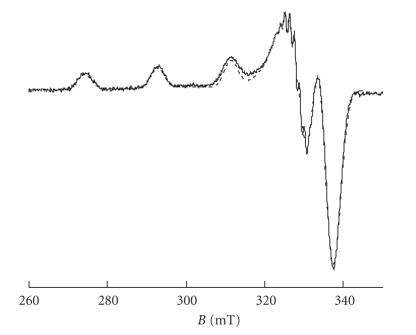
X band EPR spectrum of **1** in H_2_O/glycerol (10%), plain line: EPR spectrum, dotted line: simulation. EPR measurement conditions: temperature: 130 K, microwave frequency: 9.45 GHz, microwave power 20 mW: modulation frequency: 100 kHz, modulation amplitude: 2 G.

The X-band EPR spectra of the complexes were recorded at 130 K in a water/glycerol (9/1) or in MeOH frozen solutions. They are characteristic of Cu(II) ions in a square-planar-derived geometry in accordance with the solid state structure and indicating that **2** probably dissociates in solution give independent “Cu(ACC)_2_” units. In the case of **2**, it was not possible to distinguish two sets of parameters accounting for the two different coordination modes (*cis* or *trans* ) of the ACC ligands. The EPR spectrum of **1** is shown on Figure 3 as well as the simulated spectrum.

The simulations were performed and allowed in both cases to distinguish two distinct hyperfine coupling constants with the nitrogen atoms of the ligands (see Table 4). One coupling constant (2) is probably accounting for the two nitrogens of the bipyridine and the second (1) for the nitrogen of the ACC ligand. This indicates that the structure of the complex, and more precisely the bidentate coordination of ACC, is conserved in solution. A good simulation of **1** requires the use of a rhombic symmetry.

### 3.3. Ethylene production

**Figure 4:**
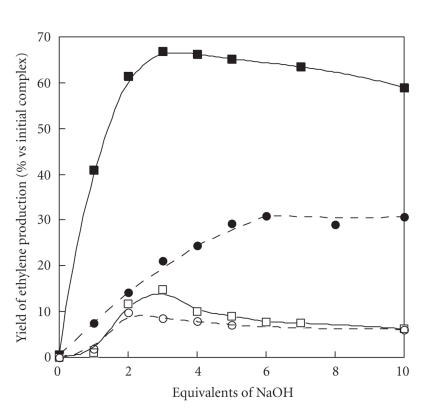
Ethylene production by complex **1** (1 mM) in the presence of 10 equivalents of H_2_O_2_ as a function of NaOH added in water (- -•- -) or in methanol (—▪—) and by a mixture of (CuClO_4_ · 6H_2_O + ACC) either in water (- -○- -) or in methanol (—□—).

ACC conversion into ethylene by complex **1** in water and in methanol was measured in the presence of 10 equivalents of hydrogen peroxide. A 1 mM solution of complex **1** was placed in a hermetically sealed tube at 20^°^C and ethylene production was measured after addition of 10 equivalents of hydrogen peroxide. It appears that ethylene production is highly dependent on the presence of base (NaOH). Figure 4 plots the ethylene production *versus* the equivalents of NaOH added in both solvents. ACC conversion reaches ca. 70% in methanol with 3 equivalents of NaOH and ca. 30% in H_2_O with 4-5 equivalents of NaOH. A similar effect was observed when Et_3_N was used instead of NaOH. These results were compared to the ethylene production of a standard solution composed of Cu(ClO_4_)_2_ · 6H_2_O at the same concentration (1 mM) in the presence of equimolar amount of ACC. Ethylene production was measured in the presence of 10 equivalents of hydrogen peroxide as a function of the base added. The results are shown on Figure 4. It appears that with this standard solution, neither in water nor in methanol, ACC conversion into ethylene reaches more than 15%. Indeed, ethylene production was 3 to 5 folds lower than with complex **1** in both solvents: in H_2_O with 6 equivalents of base, 90 nmoles of C_2_H_4_ are produced (9% conversion vs. 31% with **1**), and in MeOH with 3 equivalents of base, 140 nmoles are produced (14% conversion vs. 67% with **1**). These results emphasize the importance of controlling the coordination of the aminoacid on the metal ion. Moreover, it was verified that free ACC in the same conditions (1 mM, in H_2_O or MeOH, presence or absence of base, 10 mM of hydrogen peroxide) hardly produces ethylene, indicating that the observed activity is not due to free ACC in solution that has been released.

**Figure 5:**
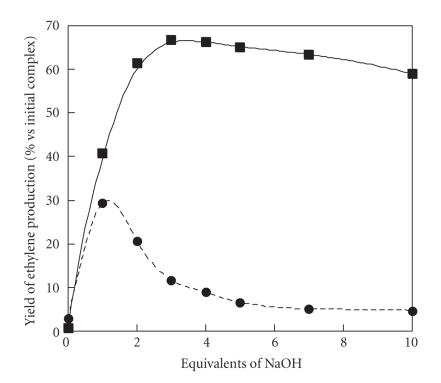
Ethylene production in MeOH by complexes **1** (—▪—) and **2** (- -•- -) in the presence of 10 equivalents of H_2_O_2_ as a function of NaOH. For complex **2**, the conversion was estimated considering only one equivalent of ACC per copper.

The ethylene production of complex **2** in the same conditions was also measured. It appears that when a 1 mM solution of complex **2** in methanol is treated by 10 equivalents of hydrogen peroxide, almost no ethylene is produced (see Figure 5). When one equivalent of base (NaOH) is added, the production reaches a maximum at ca. 30%. The conversion yield is expressed versus one ACC molecule in order to compare with complex **1** (in fact, the second ACC molecule is thus considered as the exogenic ligand). Increasing the amount of base leads to a diminution of the production. It appears that ethylene production with complex **2** is always much smaller than with complex **1**. Similar results were also observed in water suggesting that the exogenic ligand is an important factor in controlling the reactivity.

**Figure 6:**
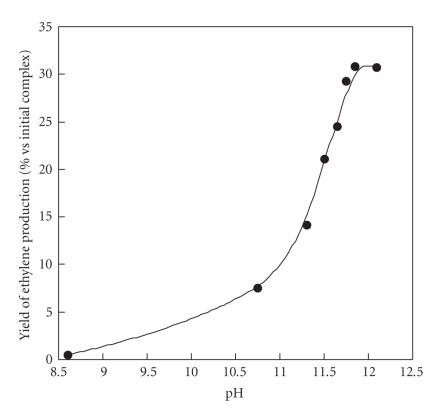
Ethylene produced by complex **1** in water as a function of pH. The pH was adjusted by addition of NaOH.

Several hypotheses can be considered for the role of the base. One can first think of a modification of the initial complex **1** in the presence of base. However, no spectral changes were observed upon addition of 1 to 5 equivalents of base on **1** neither in methanol nor in water. In particular, no changes were detected in the frozen solution X-band EPR spectrum, suggesting that the complex is poorly affected by the presence of base. A second hypothesis is that the addition of base helps to deprotonate the hydrogen peroxide. We have measured the ethylene production by complex **1** as a function of pH in water. The results are shown on Figure 6 and it appears that ethylene production reaches a maximum after pH = 11.8 (5 equivalents of base) which roughly corresponds to the pKa of hydrogen peroxide (pKa = 11.6). Adding more than 6 equivalents of NaOH, the pH increases very slowly and tends towards 12.1 and the activity is rather stable. Thus, the production of the deprotonated form of hydrogen peroxide (HOO^−^) would be important for the reaction. Studies are going on to better understand the role of the base and the reaction mechanism.

### 3.4. Detection of a reaction intermediate

**Figure 7:**
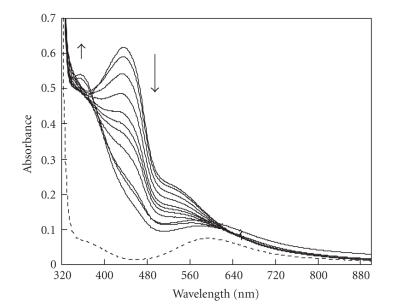
UV-visible spectra of 1mM solution of **1** in MeOH with 1 equivalents of NaOH at −20^°^C: (- - -) initial complex (—) decay of the brown intermediate versus time after addition of 10 equivalents H_2_O_2_.

At low temperature (−20^°^C in methanol), the addition of 10 equivalents of hydrogen peroxide on a solution of **1** in the presence of a few equivalents of base is followed by the appearance of a brown coloration stable a few minutes at low temperature that is characterized by an absorption band centered at 433 nm. This band decays within a few minutes to give rise to a green solution with an absorption band centered at 360 nm. Figure 7 shows the evolution of the UV-visible spectra upon addition of H_2_O_2_.

## 4. DISCUSSION AND CONCLUSION

We have thus prepared two copper-ACC complexes that were analyzed by X-Ray diffraction technique and that are able to produce ethylene from the bound ACC moiety in the presence of an excess of hydrogen peroxide. This ACC Oxidase-like activity is dependant on the presence of an exogenic ligand. At the first place, this external ligand may help to avoid rapid precipitation of copper hydroxide in basic medium. Recent results suggest however that this external ligand is also involved in the stabilization of a reaction intermediate [21]. The reactivity is also strongly dependent on the presence of a few equivalents of base and it seems that it is involved in the deprotonation of hydrogen peroxide.

During the course of the reaction, we could detect a brown intermediate formed by addition of hydrogen peroxide on **1** in the presence of a few equivalents of base. This intermediate has an absorption band centered at 433 nm and is stable a few minutes at −20^°^C. Capdevielle and Maumy observed a similar brown coloration when copper(II) salts were treated by large amount of hydrogen peroxide [22]. The brown complex was isolated and described as an oxidizing agent for several substrates. The raw formula of the intermediate was found to be CuO_2_H but its exact structure was never completely elucidated. In our case, the brown intermediate observed could be of a similar nature and it could contain a copper-peroxide moiety. Several copper-peroxo species were reported and characterized [23]. However, within the different adducts Cu/O_2_ 1 : 1 or 2 : 1, no species could correspond to our brown intermediate with an absorption band at 433 nm. Investigations are in progress to determine the nature of the intermediate and its implication in the oxidation of ACC into ethylene.

In this work, the functional and structural models are copper complexes whereas ACC Oxidase is an iron-containing enzyme. It is thus legitimate to wonder whether the results and the catalytic mechanism will be comparable to the natural system. The influence of the nature of the metal ion in the active site of a metalloprotein is the matter of actual debate. This debate is even more emphasize since within the enzymatic members of the cupin superfamily of enzymes [24, 25] to which ACCO belongs, the variety of biochemical function is provided by variations of residues in the active site and of the metal ion. Furthermore, recently, within this cupin superfamily, quercetin 2,3-dioxygenase (quercetinase) *from Bacillus subtilis* was found to be active with different metals in the active site (copper, iron, manganese, and cobalt) with best activity with Mn(II) ions [26, 27]. Interestingly, while the reaction catalyzed by the fungal quercetinases is the same as by bacterial one and the residues near the active site are highly conserved, fungal quercetinases are described as copper-containing enzymes [28]. The present work and the perpective of elucidating the mechanism by which ACC is oxidized into ethylene by the copper complexes thus open a door towards the understanding of structure-activity relationships within the dioxygen activation pathways by metal-containing systems.

## Figures and Tables

**Scheme 1:**
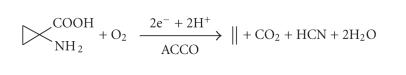
Reaction catalyzed by ACCO.

**Scheme 2:**
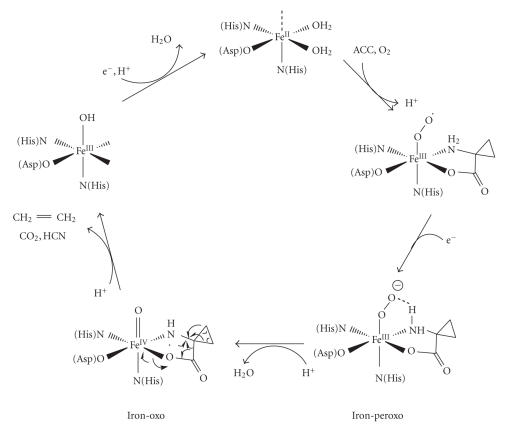
Possible catalytic mechanism for ACCO.

**Table 1:** Selected crystallographic data for [(bpy)Cu(ACC)(H_2_O)] · ClO_4_ (**1**) and [Cu(ACC)_2_ ]_3_ · 4H_2_O (**2**).

	**1**	**2**
Formula	C_14_H_16_N_3_CuClO_7_	C_24_H_44_N_6_Cu_3_O_16_
Molecular weight	437.29	863.28

Crystal size (mm)	0.4 × 0.2 × 0.2	0.3 × 0.2 × 0.1
Crystal color	blue	blue
Crystal system	Monoclinic	Monoclinic
Space group	P 2_1_/c	P 2_1_/c

Unit cell dimensions		
a (Å)	12.0940(1)	10.1510(2)
b (Å)	19.5096(3)	17.1270(2)
c (Å)	7.5502(2)	20.4500(3)
*β* (°)	102.8066(7)	101.3950(6)

V (Å^3^)	1737.14(6)	3485.28(9)
Z	4	4
T(K)	293(2)	293(2)
d (g · cm^−3^)	1.672	1.645
*λ* (Mo K*α*) (Å)	0.71073	0.71073
*μ*(Mo) (mm^−1^)	1.454	1.892
F(000)	852	1780
Reflections unique	3586	8103
Reflections observed (l *>* 2*σ*(l))	3265	6944
Parameters refined	244	442
R^a^(observed data)	0.0478	0.0390
R${}^{\text{b}}$_w_(all data)	0.1148	0.1214
Goodness-of-fit on F^2^	1.083	0.990
Largest difference peak and hole (e Å^−3^)	0.787, −0.579	0.764, −0.467

^a^ $\text{R}=\sum \left|\left|{\text{F}}_{\text{o}}\right|-\left|{\text{F}}_{\text{c}}\right|\right|/\sum \left|{\text{F}}_{\text{o}}\right|$

^b^ $w=1/\left[\sigma^{2}\left({\text{F}}_{\text{o}}^{\text{2}}\right)+{\left(\text{AP}\right)}^{2}+\text{BP}\right]$, where $\text{P}=\left({\text{F}}_{\text{o}}^{\text{2}}+2{\text{F}}_{\text{c}}^{\text{2}}\right)/3$, where A=0.0465 or 0.0465 and B=2.5576 or 2.1014 for **1** and **2**, respectively.

**Table 2:** Selected bond lengths (Å) and angles (°) for [(bpy)Cu(ACC)(H_2_O)]ClO_4_ (**1**)

*Bond lengths*	
Cu1−N1	2.005(2)
Cu1−N2	1.985(3)
Cu1−N3	2.011(3)
Cu1−O2	1.916(2)
Cu1−O13	2.421(3)
C3−C4	1.483(6)

*Angles*	

O2−Cu1−N1	84.92(9)
O2−Cu1−N2	93.00(10)
N2−Cu1−N3	81.52(10)
N3−Cu1−N1	100.51(10)
N1−Cu1−O13	102.15(9)
N2−Cu1−O13	88.69(10)
N3−Cu1−O13	83.93(9)
O2−Cu1−O13	95.73(10)
C1−C2−N1	113.2(2)
C3−C2−C4	59.2(3)

**Table 3:** EPR constants for 1 and 2 in MeOH or in water/glycerol obtained from the simulation. Coupling constants are expressed in 10^−4^ T. The contribution of naturally abundant ^63^Cu and ^65^Cu is considered, but here the values refer to ^63^Cu.

**1** (H_2_O/glycerol 10%)		**1** (MeOH)		**2** (MeOH)	
$g_{x}$ = 2.0495	${\text{A}}_{\text{Cu}}$ = 19.5	$g_{x}$ = 2.0503	${\text{A}}_{\text{Cu}}$ = 19.9	$g_{⊥}$ = 2.0499	${\text{A}}_{\text{Cu}}$ = 24.2
	${\text{A}}_{\text{N}\left(\text{2}\right)}$ = 11.3		${\text{A}}_{\text{N}\left(\text{2}\right)}$ = 13.5		${\text{A}}_{\text{N}\left(\text{2}\right)}$ = 13.5
	${\text{A}}_{\text{N}\left(\text{1}\right)}$ = 6.4		${\text{A}}_{\text{N}\left(\text{1}\right)}$ = 5.5		${\text{A}}_{\text{N}\left(\text{1}\right)}$ = 5.5

$g_{y}$ = 2.0503	${\text{A}}_{\text{Cu}}$ = 16.8	$g_{y}$ = 2.0508	${\text{A}}_{\text{Cu}}$ = 18.5	**g_//_** = 2.2507	${\text{A}}_{\text{Cu}}$ = 175.2
	${\text{A}}_{\text{N}\left(\text{2}\right)}$ = 11.3		${\text{A}}_{\text{N}\left(\text{2}\right)}$ = 13.5		${\text{A}}_{\text{N}\left(\text{2}\right)}$ = 6.5
	${\text{A}}_{\text{N}\left(\text{1}\right)}$ = 6.9		${\text{A}}_{\text{N}\left(\text{1}\right)}$ = 5.5		${\text{A}}_{\text{N}\left(\text{1}\right)}$ = 14.5

$g_{z}$ = 2.2370	${\text{A}}_{\text{Cu}}$ = 183	$g_{z}$ = 2.2325	${\text{A}}_{\text{Cu}}$ = 184	—	—
	${\text{A}}_{\text{N}\left(\text{2}\right)}$ = 6.9		${\text{A}}_{\text{N}\left(\text{2}\right)}$ = 6.5	—	—
	${\text{A}}_{\text{N}\left(\text{1}\right)}$ = 9.2		${\text{A}}_{\text{N}\left(\text{1}\right)}$ = 14.6	—	—

**Table 4:** Selected bond lengths (Å) and angles (°) for [Cu(ACC)_2_]_3_ · 4H_2_O (**2**)

*Bond lengths*	*Cis units*
Cu1−O2	1.9407(16)
Cu1−N5	2.004(2)
Cu1−O3	1.9630(16)
Cu1−N6	1.9979(19)
Cu1−O7	2.3092(18)
C29−C30	1.474(4)
C25−C26	1.473(5)

	*Trans unit*

Cu2−O8	1.9537(17)
Cu2−N12	1.990(2)
Cu2−O9	1.9590(17)
Cu2−N11	1.9817(19)
C33−C34	1.474(5)
C37−C38	1.458(5)

*Angles*	*Cis units*

O3−Cu1−N6	84.47(7)
N6−Cu1−N5	99.53(8)
N5−Cu1−O2	83.26(8)
O2−Cu1−O3	90.40(7)
N6−Cu1−O7	91.50(8)
N5−Cu1−O7	114.53(8)
O2−Cu1−O7	92.96(7)
O3−Cu1−O7	90.09(7)
N6−C28−C27	113.39(18)
C30−C28−C29	58.65(19)
N5−C24−C23	111.97(19)
C25−C24−C26	58.8(2)

	*Trans unit*

O8−Cu2−N12	84.09(8)
O8−Cu2−N11	95.72(7)
O9−Cu2−N11	84.02(8)
O9−Cu2−N12	96.12(8)
N11−C36−C35	112.36(18)
C37−C36−C38	58.1(2)
N12−C32−C31	112.59(19)
C33−C32−C34	58.7(2)
